# Supplementary material for: Treatment and Outcomes of Radiation-Induced Soft Tissue Sarcomas of the Extremities and Trunk—A Systematic Review of the Literature
Source: Cancers (Basel). 2023 Nov 25;15(23):5584. doi: 10.3390/cancers15235584 (PMC10705150; doi:10.3390/cancers15235584)
Supplement: Supplementary file 1 [file cancers-15-05584-s001.zip › cancers-2714362-supplementary.pdf]

Supplementary Table S1. Additional information on included studies.

First Author	Year	Title	Country	Institution	Study Design
**Kao et al. [11]**	2023	Clinical and Pathologic Characterization of 94 Radiation-Associated Sarcomas: Our Institutional Experience	USA	University of Washington	Retrospective Cohort
**Spalek et al. [3]**	2021	The Management of Radiation-Induced Sarcomas: A Cohort Analysis from a Sarcoma Tertiary Center	Poland	Maria Sklodowska-Curie National Research Institute of Oncology	Retrospective Cohort
**Callesen et al. [20]**	2020	Radiation-Induced Sarcoma: A Retrospective Population-Based Study Over 34 Years in a Single Institution	Denmark	Aarhus University Hospital	Retrospective Cohort
**Italiano et al. [10]**	2018	Patterns of Care and Outcome Radiation-Induced Soft Tissue Sarcomas	France	Institut Bergonié	Retrospective Cohort
**Joo et al. [26]**	2018	Post-radiation sarcoma: A study by the Eastern Asian Musculoskeletal Oncology Group	Korea	St. Vincent's Hospital	Retrospective Cohort
**Dineen et al. [23]**	2015	Radiation-Associated Undifferentiated Pleomorphic Sarcoma is Associated with Worse Clinical Outcomes than Sporadic Lesions	USA	UT MD Anderson Cancer Center	Retrospective Cohort
**Kim et al. [27]**	2015	Radiation-Induced Sarcoma: A 15-Year Experience in a Single Large Tertiary Referral Center	Korea	Seoul National University	Retrospective Cohort
**Riad et al. [4]**	2012	The Clinical and Functional Outcome for Patients With Radiation-Induced Soft Tissue Sarcoma	Canada	Mount Sinai Hospital, Ottawa General Hospital, McGill University Health Centre, Vanderbilt Medical Center	Retrospective Cohort
**Gladdy et al. [5]**	2010	Do Radiation-Associated Soft Tissue Sarcomas Have the Same Prognosis As Sporadic Soft Tissue Sarcomas?	USA	Memorial Sloan-Kettering Cancer Center	Retrospective Cohort
**Neuhaus et al. [12]**	2009	Treatment and outcome of radiation-induced soft-tissue sarcomas at a specialist institution	UK	Royal Marsden Hospital	Retrospective Cohort
**Holt et al. [24]**	2006	Multifocality and Multifocal Postradiation Sarcomas	USA, Canada	Vanderbilt University Medical Center, Mount Sinai Hospital, Ortho Indianapolis	Retrospective Cohort
**Thijssens et al. [7]**	2005	Radiation-Induced Sarcoma: A Challenge for the Surgeon	The Netherlands	Groningen University Medical Centre	Retrospective Cohort
**Cha et al. [21]**	2004	Long-term Results With Resection of Radiation-Induced Soft Tissue Sarcomas	USA	Memorial Sloan-Kettering Cancer Center	Retrospective Cohort
**Fang et al. [6]**	2004	Postradiation soft tissue sarcoma: a multiinstitutional analysis of 14 cases in Japan	Japan	Cancer Institute Hospital	Retrospective Cohort

**Lagrange et al. [9]**	2000	Sarcoma after Radiation Therapy: Retrospective Multiinstitutional Study of 80 Histologically Confirmed Cases	France	Fédération Nationale des Centres de Lutte Contre le Cancer	Retrospective Cohort
**Inoue et al. [25]**	2000	Clinicopathologic Features and Treatment of Postirradiation Sarcoma of Bone and Soft Tissue	USA	Mayo Clinic	Retrospective Cohort
**Bloechle et al. [18]**	1995	Post-irradiation Soft Tissue Sarcoma	Germany	University Hospital Eppendorf	Retrospective Cohort
**Brady et al. [19]**	1992	Radiation-Associated Sarcoma of Bone and Sof Tissue	USA	Memorial Sloan-Kettering Cancer Center	Retrospective Cohort
**Wiklund et al. [8]**	1991	Postirradiation Sarcoma	Finland	Helsinki University Central Hospital	Retrospective Cohort
**Laskin et al. [28]**	1988	Postradiation Soft Tissue Sarcomas	USA	Armed Forces Institute of Pathology	Retrospective Cohort
**Davidson et al. [22]**	1986	Radiation-induced soft-tissue sarcoma	UK	Royal Marsden Hospital	Retrospective Cohort
